# Functional Neuroimaging Correlates of Burnout among Internal Medicine Residents and Faculty Members

**DOI:** 10.3389/fpsyt.2013.00131

**Published:** 2013-10-15

**Authors:** Steven J. Durning, Michelle Costanzo, Anthony R. Artino, Liselotte N. Dyrbye, Thomas J. Beckman, Lambert Schuwirth, Eric Holmboe, Michael J. Roy, Christopher M. Wittich, Rebecca S. Lipner, Cees van der Vleuten

**Affiliations:** ^1^Department of Medicine, Uniformed Services University of the Health Sciences, Bethesda, MD, USA; ^2^Mayo Clinic, Rochester, MN, USA; ^3^School of Medicine, Flinders University, Bedford Park, SA, Australia; ^4^American Board of Internal Medicine, Philadelphia, PA, USA; ^5^Department of Educational Development and Research, Maastricht University, Maastricht, Netherlands

**Keywords:** expertise, burnout, clinical reasoning, cognitive load, fMRI

## Abstract

Burnout is prevalent in residency training and practice and is linked to medical error and suboptimal patient care. However, little is known about how burnout affects clinical reasoning, which is essential to safe and effective care. The aim of this study was to examine how burnout modulates brain activity during clinical reasoning in physicians. Using functional Magnetic Resonance Imaging (fMRI), brain activity was assessed in internal medicine residents (*n* = 10) and board-certified internists (faculty, *n* = 17) from the Uniformed Services University (USUHS) while they answered and reflected upon United States Medical Licensing Examination and American Board of Internal Medicine multiple-choice questions. Participants also completed a validated two-item burnout scale, which includes an item assessing emotional exhaustion and an item assessing depersonalization. Whole brain covariate analysis was used to examine blood-oxygen-level-dependent (BOLD) signal during answering and reflecting upon clinical problems with respect to burnout scores. Higher depersonalization scores were associated with less BOLD signal in the right dorsolateral prefrontal cortex and middle frontal gyrus during reflecting on clinical problems and less BOLD signal in the bilateral precuneus while answering clinical problems in residents. Higher emotional exhaustion scores were associated with more right posterior cingulate cortex and middle frontal gyrus BOLD signal in residents. Examination of faculty revealed no significant influence of burnout on brain activity. Residents appear to be more susceptible to burnout effects on clinical reasoning, which may indicate that residents may need both cognitive and emotional support to improve quality of life and to optimize performance and learning. These results inform our understanding of mental stress, cognitive control as well as cognitive load theory.

## Introduction

Burnout is a syndrome characterized by emotional exhaustion, depersonalization, and a low sense of personal accomplishment (1). Burnout has been identified in 40–50% of physicians (2), residents (3, 4), and medical students (5, 6). It is believed that recurrent stressful interactions with patients lead to high emotional exhaustion and depersonalization (e.g., burnout). This high prevalence is alarming as multiple studies have found that burnout can negatively impact self-reported quality of care rendered to patients (7–11). Longitudinal studies involving medical residents further suggests that burnout predicts subsequent self-reported medical error (8, 10). These findings coupled with a recent national study of nearly all U.S. medical residents that demonstrated substantially worse performance on a standardized medical knowledge assessment among learners with high emotional exhaustion (4) and suggested that burnout may impede cognitive performance.

The well-documented detrimental impact of burnout on physicians and consequent self-reported patient care indicates the need for measures to prevent its negative effects. Validated measures to determine burnout in physicians exist. The gold standard measure of burnout is the Maslach Burnout Inventory (MBI) (1). This measure, while providing a means to detect the phenomenon of burnout, does not provide insight into the impact it might have on a physician’s clinical reasoning (the thought processes up to and including arriving at a diagnosis and treatment for a patient). Such a mechanistic understanding can help medical educators structure and implement detection, monitoring, and remediation of burnout.

Previous studies in a non-medical population indicate that cognitive control is compromised by burnout (12, 13). Cognitive control is experience-dependent, and encompasses information-processing ability, attention allocation, and resultant behavior (14). The prefrontal cortex is central to this process, integrating sensory information, goals and rules, and influencing the coordination of behavior via temporal, parietal, and motor cortices (14). Deficits in cognitive control can manifest as limitations in attention, memory, and comprehension, which are all relevant to clinical reasoning. In addition, studies have reported that excessive work demands and loss of autonomy at work contribute to burnout (15, 16). Thus, stress-related compromised cognitive function may explain the underlying basis of burnout-dependent medical errors in patient care.

Neurobiologically, mental stress compromises cognitive function by producing changes in prefrontal regions including the dorsolateral prefrontal cortex (DLPFC) (14, 17, 18). Such changes in the prefrontal cortex are reversible (17), and suggest a neural mechanism for the effect of burnout on clinical reasoning and subsequent medical errors. Thus, investigating the neurobiological impact of burnout on physicians’ reasoning may reveal important insights into how workplace stress influences clinical reasoning performance.

The aim of this study was to examine how burnout modulates brain activity during clinical reasoning in physicians. Solving and reflecting upon clinical problems involves the ability to control and evaluate cognitive processes (19). We hypothesize that increases in emotional exhaustion and depersonalization – the key components of burnout among physicians – will be associated with a relative reduction in blood-oxygen-level-dependent (BOLD) signal in cognitive control regions, particularly the DLPFC. We further hypothesize that this relationship between burnout and these regions may be most apparent in more novice learners (residents), rather than experienced physicians (faculty) who have greater cognitive control due to more clinical experience (e.g., better engrained illness scripts, more robust cognitive strategies) which makes their clinical reasoning less vulnerable to the effects of burnout.

## Materials and Methods

### Participants

Following informed consent, board-certified internal medicine attending physicians with faculty appointments and internal medicine residents at the Uniformed Services University (USUHS) participated in the study. Faculty and residents were recruited by email invitation and no compensation was provided with participation in this study. The mean age of the board-certified internists (faculty) was 39.5 ± 7 (range = 32–51 years), and there were 15 men and 2 women. For the internal medicine residents, the mean age was 29.6 ± 2 (range 28–35 years), and there were 10 men and 7 women. Exclusion criteria were the presence of shrapnel or surgical metal devices, inability to complete an fMRI due to anxiety or claustrophobia, the daily use of calcium channel blockers (which can impact regional blood flow), or pregnancy. The Institutional Review Boards of the USUHS and Walter Reed Army Medical Center approved the study.

### Measurements

#### Burnout

Just prior to entering the scanner, each participant completed two single-item measures adapted from the MBI (1). Multiple independent samples of over 10,000 physicians and medical students have previously shown that these two items (one measuring emotional exhaustion and the other measuring depersonalization which are summed for a total burnout score) effectively stratify the risk of burnout (20, 21). Previous studies have also reported that the areas under the receiver operating characteristic curve for the emotional exhaustion and depersonalization single-items in comparison to the full MBI domain scores were 0.94 and 0.93, respectively, and the positive predictive values of these single-item thresholds for high levels of emotional exhaustion and depersonalization were 88.2 and 89.6%, respectively (20, 21).

#### Functional magnetic resonance imaging

Acquisitions were performed using an echo-planar imaging (EPI) sequence of 40 contiguous sagittal slices per brain volume (TR = 2000 ms, TE = 25 ms, flip angle = 60, slice thickness = 4.0 mm). In-plane resolution was 3.75 + 3.75 mm (64 × 64 voxels). During the imaging session, a high-resolution T1-weighted image was acquired for anatomical reference (three-dimensional GRE; TR = 6.6 ms, TE = 2.5 ms, flip angle = 12°). This image consisted of 312 sagittal slices with a slice thickness of 0.6 mm and an in-plane resolution of 0.468 × 0.468 mm (512 × 512 voxels).

### Clinical reasoning task

Participants answered validated MCQs from the American Board of Internal Medicine (ABIM) and National Board of Medical Examiners (NBME). These organizations are responsible for certifying or licensing physicians in the U.S., and they validate the appropriateness of their items by subjecting them to a rigorous internal content review and performance analysis. Multiple-choice items are particularly well suited for assessment of clinical reasoning performance while in the fMRI scanner as participants are able to make selections via handheld buttons as opposed to having to speak (jaw motion impairs fMRI image interpretation).

Participants answered 32 questions: 16 NBME items [United States Medical Licensing Examination (USMLE) Step 2 Clinical Knowledge items] and 16 ABIM items [Maintenance of Certification (MOC) MCQ]. Items that required the integration and synthesis of data, queried “What is the most likely diagnosis?” (i.e., the examination items assessed clinical reasoning), did not include images, and had favorable item statistics based on their use in thousands of participants, within the content areas of cardiology and rheumatology were selected.

Each MCQ was projected in three phases: reading, answering, and reflecting. In the reading phase, the stem (question) appeared ending with “what is the most likely diagnosis?” or a related diagnostic question, but the answer options were not displayed. Each participant was given 60 s to read the stem and push any button to move on to the answer options (the second or “answering” phase). Participants were then given 7 s to choose an answer option using the provided handheld response buttons. The final phase (“reflection” phase) was then entered, where participants were instructed to reflect on how they arrived at the diagnosis (“how did you establish the diagnosis for this item”) without actually speaking. This phase lasted 14 s. The times given for reading, answering, and reflecting were based upon national standards for answering these multiple-choice questions (1 min per question for high stakes examinations). We compared the phases (answering relative to reading, reflecting relative to reading) to elicit task-specific differences, each employing different aspects of the construct of reasoning and requiring cognitive control. Participants were not aware of the study hypotheses.

### Functional magnetic resonance imaging data analysis

All fMRI data were processed using the AFNI software package in accordance with previously published methods (22, 23). The participant’s EPI scans were preprocessed by first removing the three volumes (6 s) from each 4D time series. Next the scans were corrected for slice timing and motion then co-registered to the T1 anatomical image (anatomic scans were registered to Talairach space). Next the images were spatially smoothed using 8 mm full width at half-maximum Gaussian kernel and converted to percent-change-from-mean.

For the first level analysis, the four data sets for each subject were concatenated and the general linear model (GLM) was used to compute a voxel-based measure of the effects of interest (24). Hemodynamic response estimates were modeled for the answering and reflecting question phases, and the reading phase was used as the baseline. The “answer” times varied from question to question (depending on how quickly the participant answered) and were modeled with a gamma-variate function with variable duration and variable relative amplitude (amplitude variation was based on duration variation). The “reflection” time was constant at 14 s and was modeled with a non-variable gamma-variate. The GLM analysis determined the significance of these model time-courses, along with head motion parameters, to generate β coefficients and *t* statistics for each voxel, for the contrasts of interest: answer phase relative to reading or answer contrast (answer > reading), and reflection phase relative to reading or reflection contrast (reflecting > reading).

For the covariate analysis, subject-specific BOLD signal intensity changes (β coefficients) for answering and reflection contrasts were used in the second-level group analysis. The answering contrast (answer > reading) and reflection contrast (reflection > reading) images were entered into the *t*-test program in AFNI, 3dttest++, to examine voxel-wise covariates for each subject. The program computed a regression for each voxel, to estimate the mean and the slopes of the contrast data (difference between the conditions) with respect to variations in the covariates. The covariates of interest included the emotional exhaustion (one item from two item well-being index), depersonalization (one item from two item well-being index), and the burnout composite score (total score on two item well-being index). Results of the second-level analyses were corrected for multiple comparisons using family-wise error (FWE) correction (from a Monte Carlo simulations using AFNI’s 3dClustSim). This produced corrected *p* values (*p* < 0.05) based on cluster size. The presented data represents the covariates effect on the difference between the condition (slope) and the *t*-statistic of this slope.

## Results

### Burnout

The paired *t*-test revealed that the composite burnout measure was significantly different between the groups (*p* = 0.015), with the faculty reporting a mean score of 2.467 (SD 2.26) and residents reporting a mean value of 5.33 (SD 3.84); this group difference represents a large effect (Cohen’s *d* = 0.94) and is consistent with larger studies demonstrating higher burnout in residents than in attending physicians (Table 1).

**Table 1:** **Group demographics and Maslach Burnout Inventory scores**.

Group	Age	Male	Female	Emotional exhaustion	Depersonalization	Composite
Internal medicine residents	29.6 ± 2	10	7	3 (SD 2.12)	2.33 (SD 2)	5.33 (SD 3.84)
Board-certified internists (faculty)	39.5 ± 7	15	2	1.67 (SD 1.29)	0.8 (SD 1.08)	2.467 (SD 2.26)

Summary of demographics and burnout scores (emotional exhaustion “I feel burned out from my work;” depersonalization “I have become more callous toward people since I took this job;” and composite scores) are reported.

Emotional exhaustion was significantly different between the groups (*p* = 0.033), with the faculty reporting a mean score of 1.67 (SD 1.29) and residents reporting a mean value of 3 (SD 2.12); again, a large effect (Cohen’s *d* = 0.80). Depersonalization was also significantly different between the groups (*p* = 0.011), with the faculty reporting a mean score of 0.8 (SD 1.08) compared to the residents’ mean value of 2.33 (SD 2); again, a large effect (Cohen’s *d* = 1.04). Greater depersonalization scores in residents (vs. faculty) are likewise consistent with larger studies of resident and attending physicians (Table 1).

### Functional magnetic resonance imaging

#### Faculty

Covariate analysis of well-being measures of emotional exhaustion, depersonalization, and overall burnout revealed no significant correlation with BOLD signal for the answering and reflecting contrasts among faculty, suggesting no relationship between degree of burnout and activity in cognitive control regions (i.e., the prefrontal cortex).

#### Residents

**Figure 1:**
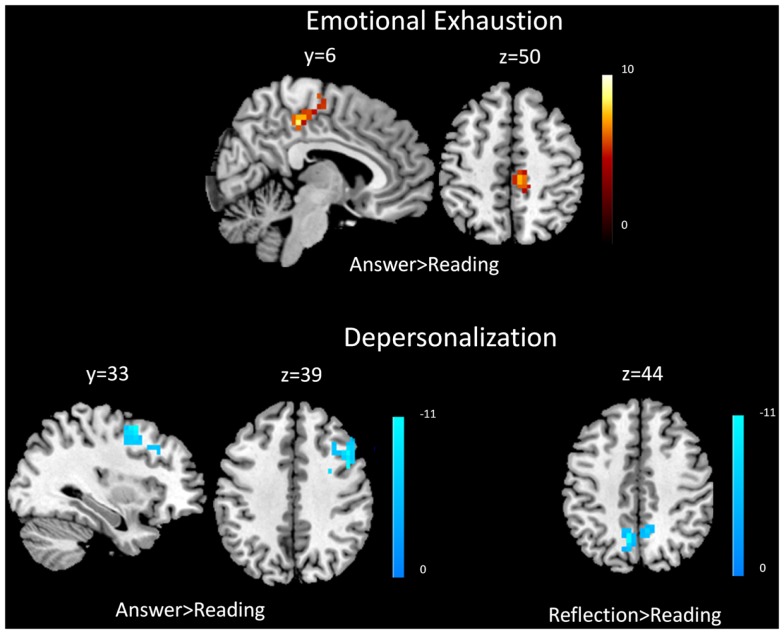
**Functional magnetic resonance imaging results**. Covariate results for emotional exhaustion and the beta values of the answering > reading contrast (top panel) for residents. Covariate results for depersonalization and answering > reading contrast (left) and reflection > reading contrast (right) for residents (bottom panel). The color bar denotes the *t*-scores of the estimated slope of the beta values vs. the covariate.

Covariate analysis revealed increased BOLD signal in the right middle frontal gyrus and right posterior cingulate cortex with respect to self-reported emotional exhaustion (FWE correction, *p* < 0.05) when answering (answering > reading) clinical problems in the (Table 2, Figure 1). No significant relationship between emotional exhaustion and reflecting (reflection > reading) was revealed.

**Table 2:** **Covariate analysis results**.

	BA	Hemi	*t*-score	*x*	*y*	*z*	*p*
**EMOTIONAL EXHAUSTION**							
**Answer ***>*** reading**
Middle frontal gyrus	6	R	5.44	3	−15	56	0.029
Posterior cingulate cortex	31	R	11.09	12	−32	41	<0.001
**DEPERSONALIZATION**							
**Answer ***>*** reading**
Precuneus	7	L	−15.4	−8	−58	40	<0.001
		R	−9.49	10	−54	37	<0.001
**Reflecting ***>*** reading**
Middle frontal gyrus	6	R	−9.55	34	6	52	<0.001
Dorsolateral prefrontal cortex	9	R	−7.69	44	16	36	<0.001

Covariate analysis revealed increased and decreased BOLD signal with respect to self-reported burnout measures (emotional exhaustion, top; depersonalization, bottom) in residents when answering (answering > reading) and reflecting (reflecting > reading) upon clinical problems. Brodmann areas (BA), hemisphere (Hemi), Max *t*-value (*t*-score), corresponding *p* values, and Talairach coordinates (*x*, *y*, *z*) are reported.

Covariate analysis also revealed significant (FWE correction, *p* < 0.05) decreased BOLD signal in the bilateral precuneus during the answering phase (answering > reading) in relation to depersonalization. Significant (FWE correction, *p* < 0.05) decreased BOLD signal was also found during reflecting (reflection > reading) in the right middle frontal gyrus and right DLPFC with respect to depersonalization (Table 2; Figure 1).

Examination of the composite burnout score revealed significant increased BOLD signal in the right middle frontal gyrus [peak intensity location (*t*-score) at *x* = 2, *y* = −23, *z* = 55, *p* = 0.0014] and right posterior cingulate cortex (peak intensity location *x* = 11, *y* = −32, *z* = 40, *p* < 0.001) during answering (answering > reading) and decreased BOLD signal in the right DLPFC (peak intensity location *x* = 42, *y* = 16, *z* = 37, *p* = 0.0037) during reflecting (reflection > reading).

## Discussion

This is the first study to demonstrate associations between burnout, a prevalent phenomenon in physicians, and functional MRI findings. Specifically, we found that depersonalization scores are associated with reduced BOLD signal in the DLPFC, precuneus, and middle frontal gyrus, and that emotional exhaustion scores are associated with increased BOLD signal in the posterior cingulate cortex and middle frontal gyrus among resident physicians. Additionally, and consistent with the burnout literature (3, 4), we found that residents had significantly higher scores than faculty physicians on measures of depersonalization and emotional exhaustion. These findings support our hypothesis that residents are more susceptible to burnout’s effects on cognitive function than faculty, but these modulatory effects extend to brain regions beyond our hypothesized impact on the prefrontal cortex. Collectively, the findings suggest that residents may be more vulnerable to burnout-dependent changes in neural activity while solving multiple-choice questions assessing clinical reasoning. This may be due to such examinations being more emotional to this group, given the relevance of needing to pass a MCQ exam to achieve board certification. Educational theory would suggest that residents may have less cognitive reserve due to less expertise. We discuss the theory of cognitive load in more detail below. In turn, this concept of reduced cognitive reserve and the impact of additional demands on these limited cognitive resources, for example through the detrimental impact of burnout, may be an important source of medical errors related to clinical reasoning tasks in practice.

### Burnout-dependent compromised cognitive processing in residents

Consistent with our hypothesis there was decreased BOLD signal in the right DLPFC (BA9) with respect to burnout, specifically depersonalization, in residents, but only while reflecting on clinical problems. The DLPFC is involved in various cognitive functions including monitoring and managing remembered information, and implementation strategies to assist with memory (25, 26) – all processes that would be expected to aid in reflecting on and solving clinical problems. Interestingly, the right lateralization suggests a valence-related explanation, since the right frontal cortex is more dominant in processing negative affect and has been previously reported to be reduced during negative emotion content with a working memory task (27, 28). Our results support the notion that mental stress (i.e., burnout) negatively impacts the DLPFC activity during cognitive tasks, a finding consistent with previous studies reporting such an effect during working memory manipulations (18, 28). The underlying mechanisms for such stress-dependent disruption in prefrontal function may relate to catecholamine influences and changes in intracellular signaling pathways, as outlined in a recent review (29). Collectively, these findings may provide insight into burnout-related impairments in high-order cognitive function and have implications for medical education and training.

In addition to the DLPFC, our results revealed a similar negative relationship between depersonalization and the right middle frontal gyrus (BA6) with residents. This region has been demonstrated to be sensitive to emotionally unpleasant visual cues (30, 31), and is also critical to perspective-taking and empathy. The right lateralization of these findings again supports the notion of the influence of affect on cognitive processes, with the right MFG associated with avoidance motivation that in turn influences top-down control of goal-directed behavior (32). Burnout may induce change in the down-regulation of such cortical regions leading to impaired reflective processing of clinical problems that could adversely impact the quality of patient care (33, 34).

Significant decrease BOLD signal in the bilateral precuneus during answering MCQs with respect to depersonalization revealed additional insight into the neural basis of burnout-dependent changes in brain activity with residents during cognitive challenge. Emerging work suggests that the precuneus is important in non-analytic reasoning (23). Thus, it appears that depersonalization may impair pattern recognition in addition to disrupting vital prefrontal cortex activity, which appears necessary for analytic reasoning.

### Burnout-dependent increases in cognitive load in residents

Our results not only revealed functional decreases in BOLD signal with respect to burnout measures but also found evidence of selective burnout-dependent increases in BOLD signal. Cognitive load theory (CLT) can help with the interpretation of these fMRI findings (35, 36). CLT refers to limitations in human cognitive architecture; we can only hold, or process, a limited number of pieces of information (7 ± 2) in our working or short-term memory at a given time. This theory suggests that both depersonalization and emotional exhaustion could synergistically and adversely impact clinical reasoning. Our results revealed that emotional exhaustion was associated with significant increase in BOLD signal of the middle frontal gyrus and the posterior cingulate cortex during the task of answering clinical reasoning items. CLT would postulate that the impact of emotional exhaustion would further exacerbate limited processing space through inefficient activation, like a computer processing unit whose function is slowed in performing a desired task by too many simultaneous processes. Emotional exhaustion induces greater albeit inefficient brain activation, which ties up much of the brain’s limited cognitive processing capacity. Therefore, for complex tasks such as clinical reasoning, emotional exhaustion could further limit the available cognitive resources, particularly in residents. Less experienced learners would already be expected to exhibit less efficient neural activation, and our work in fact provides insight into how burnout could lead to additional inefficiency in activation. Emotional exhaustion would theoretically further reduce functional neuroactivation in already inefficiently activated areas, thus impairing clinical reasoning and potentially contributing to medical errors.

### Faculty and burnout

It was notable that faculty members’ brain activation during answering and reflecting on clinical problems did not significantly correlate with burnout measures. There are several potential explanations. It is possible that the effect of burnout is mitigated by faculty members’ greater experience-dependent cognitive control (i.e., through neural efficiency leading to more available cognitive resources) when solving clinical reasoning problems making them less vulnerable to burnout-related changes in brain function (14). Also, our residents had higher emotional exhaustion and depersonalization scores, thus these findings could be independent of expertise and suggest that elevated burnout correlates with brain activity during cognitively challenging tasks (i.e., clinical reasoning). It is quite plausible that more years of experience have enabled faculty to adapt to the impact of burnout on cognitive function, likely through enhanced automatization of clinical reasoning processes. Thus, faculty appear to require less cognitive resources, engendering resilience to exhaustion.

There are several limitations to our study. Though similar to prior neuroimaging studies of specialized groups (37–39), our sample size was relatively small and our rate of burnout was low. Our measure of burnout was based on a two-item self-report measure, which while it has been validated, it is not the full MBI. We made comparisons between reading, answering, and reflecting phases, which arguably do represent different cognitive tasks, but there is also some degree of overlap between them so that we cannot, for example prevent someone from beginning to think of an answer while reading. By only showing the possible answers during the answer phase, we attempted to mitigate this effect. There are also differences in the age (virtually unavoidable when comparing faculty and residents) and gender of our two populations, which one might make an attempt to control for in future studies. In addition, it is also possible that individuals of different age and experience, even within our resident and faculty groups, can use different cognitive processes. We did not screen participants for conditions such as depression that could affect cognition.

Our findings provide some support for resident work hour limitations, in order to mitigate emotional exhaustion and depersonalization, but further studies are needed to confirm that placement of such limits does indeed translate into improved cognitive performance and less burnout. It is notable that restrictions in work hours have not conclusively been shown to reduce burnout (40). While people might get more “rest” with fewer hours, if the hours that are spent in the hospital are highly stressful with poor emotional and cognitive support, then one would still expect substantial burnout (41, 42). Our study finds reason for concern with regard to neurological functions necessary for physician work and learning. Future research could also more carefully assess junior and senior residents to try to pinpoint when significant expertise, and consequent resilience, is obtained, which would have particular relevance for the structuring of residency training programs and work hour limits. Investigations targeting how to reduce burnout, as well as helping residents recover from burnout to reduce the cognitive impact, are needed. Our findings also support providing residents with stress coping strategies.

### Conclusion

This is the first study to explore the neuroimaging correlates of burnout in residents and attending physicians while performing a “gold standard” for physician performance. Burnout was associated with neuroimaging changes in residents that is consistent with, and further informs, our understanding of cognitive control and CLT. Residents appear to be more susceptible to burnout effects on cognition, which indicates that residents need both cognitive and emotional support in order to work and learn. To reduce medical error and improve quality of care, efforts are needed both to prevent burnout and to develop strategies to facilitate recovery from burnout in physicians in training.

## Conflict of Interest Statement

The authors declare that the research was conducted in the absence of any commercial or financial relationships that could be construed as a potential conflict of interest.
